# Broadening diversity, equity, accessibility, and inclusion in the process and development of climate assessments

**DOI:** 10.1007/s10584-025-03873-z

**Published:** 2025-03-20

**Authors:** Eric K. Chu, Gillian Bowser, Abby G. Frazier, Alyssa Quintyne, Linda Shi, Pamela McElwee

**Affiliations:** 1 Present Address: Department of Human Ecology, University of California, Davis, Davis, CA USA; 2 Warner College of Natural Resources, Colorado State University, Fort Collins, CO USA; 3 Graduate School of Geography, Clark University, Worcester, MA USA; 4 Community Organizer, Fairbanks, AK USA; 5 Department of City and Regional Planning, Cornell University, Ithaca, NY USA; 6 School of Environmental and Biological Sciences, Rutgers University, New Brunswick, NJ USA

**Keywords:** Climate change assessments, Diversity, Equity, Inclusion, Participation

## Abstract

Comprehensive assessments of scientific knowledge are essential to inform efforts to mitigate greenhouse gas emissions and adapt to climate change impacts. The Fifth National Climate Assessment (NCA5), released in late 2023, adopted clear diversity, equity, accessibility, and inclusion (DEAI) goals and trainings, which helped diversify expert participation, broaden the types of knowledge included, and widen public engagement. This Letter, written by NCA5 authors, reflects on the impacts and limitations of these efforts and suggests specific actions to further promote collaboration, honor and recognize the knowledge of frontline communities, and guide more just and holistic climate assessments.

## Introduction

Climate assessments operate in between science and policy while explicitly pursuing a societal goal (Oppenheimer et al. 2019). Greater author diversity – in terms of geography, career stage, gender, race, ethnicity, and academic discipline – arguably enhances the epistemological rigor of climate assessments by including a breadth of perspectives (Vardy et al. 2017). An ability to speak from a broad perspective is important if climate assessments are to produce legitimate and authoritative climate knowledge to inform policy and reflect the priorities and needs of the public (Standring 2022). The societal influence of climate assessments also depends on public perceptions of their process and the extent to which they are fair, inclusive, and rigorous (Mach and Field 2017). The authority of climate assessments is therefore derived from both their substantive legitimacy (i.e., scientific validity and robustness) and their representational legitimacy to speak from multiple knowledge spaces (Beck and Mahony 2018).

The National Climate Assessment (NCA), produced by the U.S. Global Change Research Program (USGCRP), provides policy-relevant information on how people within the United States experience climate change. The NCA identifies areas and degrees of scientific agreement and policy relevance through an authorship team consisting of academics, researchers, practitioners, and federal scientists and managers. In addition, the NCA is guided by a steering committee of federal managers and an extensive public engagement process (Avery et al. 2025). Using the Fifth National Climate Assessment (NCA5) released in late 2023 (USGCRP 2023) as an example, we explore the different enablers of diversity, equity, accessibility, and inclusion (DEAI) in developing climate assessments. There are currently questions about whether efforts like the NCA adequately synthesize the vast amounts of literature and represent the diversity of scientific disciplines (Callaghan et al. 2020). Additional critiques ask who participates in assessments, whether processes are accessible and inclusive of diverse voices, and whether assessments reflect frontline communities’ experiences (Standring 2022). Such questions are situated in a broader, ongoing conversation about diversity and representation in science (Bernard and Cooperdock 2018).

This Letter explores how the principles of DEAI, when applied to the NCA process, can help promote collaboration, honor the experiences and knowledge of frontline communities, and guide climate research that is more just and holistic. Authors of this Letter participated in the NCA5 process as chapter leads or authors. These reflections are based on our diverse experiences in leading chapter drafting efforts, spearheading DEAI initiatives throughout the assessment process, and managing cross-chapter logistics.

## Insights on DEAI from NCA5

Here, we explore efforts to further the application of DEAI to NCA5, which began development in early 2020 and was released in November 2023. We do not give a full historical account of all climate assessments as there is already extensive literature analyzing their roles, responsibilities, and policy impacts. These studies have found that assessments have a disciplinary bias towards the natural sciences and economics (Corbera et al. 2016), lack alignment with the social sciences (Victor 2015; Maxwell et al. 2022), lack inclusion of practitioners (Viner and Howarth 2014; Rigg and Mason 2018), and systematically exclude Indigenous Knowledge (Ford et al. 2016; Carmona et al. 2023). Rather, our aim is to show how the NCA5 has tried to overcome some of these barriers and to comment on the effectiveness of these efforts.

### Structuring for DEAI in NCA5’s design

Climate assessments tend to have high barriers to participation since scientific training and knowledge production processes reflect the structural inequalities found within academia and society. Systemic barriers can be especially high along gender lines and for authors who are Black, Indigenous, or persons of color (Campbell et al. 2013; Nielsen et al. 2017; Gay-Antaki and Liverman 2018). In light of these critiques and building on past NCA efforts, the NCA5 process was designed to be transparent and inclusive. The NCA5 leadership team emphasized DEAI in its work, with the goals of: (1) diverse and inclusive participation in the assessment development process; (2) integration of equity and environmental justice in NCA5 content; and (3) accessible and inclusive engagement, outreach, and communication.

NCA5 also benefited from strong leadership and guidance on defining and enabling DEAI efforts. For example, USGCRP provided authors with a clear Code of Conduct, which aimed to foster a safe, inclusive, and respectful working environment for all assessment participants. Still, NCA5 authors noted the need for a consistent definition of DEAI for the entire assessment effort, especially since some authors came from professional backgrounds or scientific disciplines where engagements with aspects of DEAI are less common. This would ensure that author teams agree on the need to elevate historically excluded perspectives — such as Indigenous Knowledge and local community knowledge — and to provide a multidisciplinary approach to assessing climate science. Therefore, there may be need for additional guidance and training for scientists who may not be as well-versed in or aware of equity and inclusion priorities.

### Diversifying NCA5 authors and contributors by geography and profession

NCA5 encouraged chapter leads to build diverse author teams, recognizing that scientific expertise can come in many forms — including across disciplinary backgrounds, professions, lived experiences, career stages, geographical locations, and demographic characteristics. However, building diverse author teams proved to be a challenge.

**Fig. 1:**
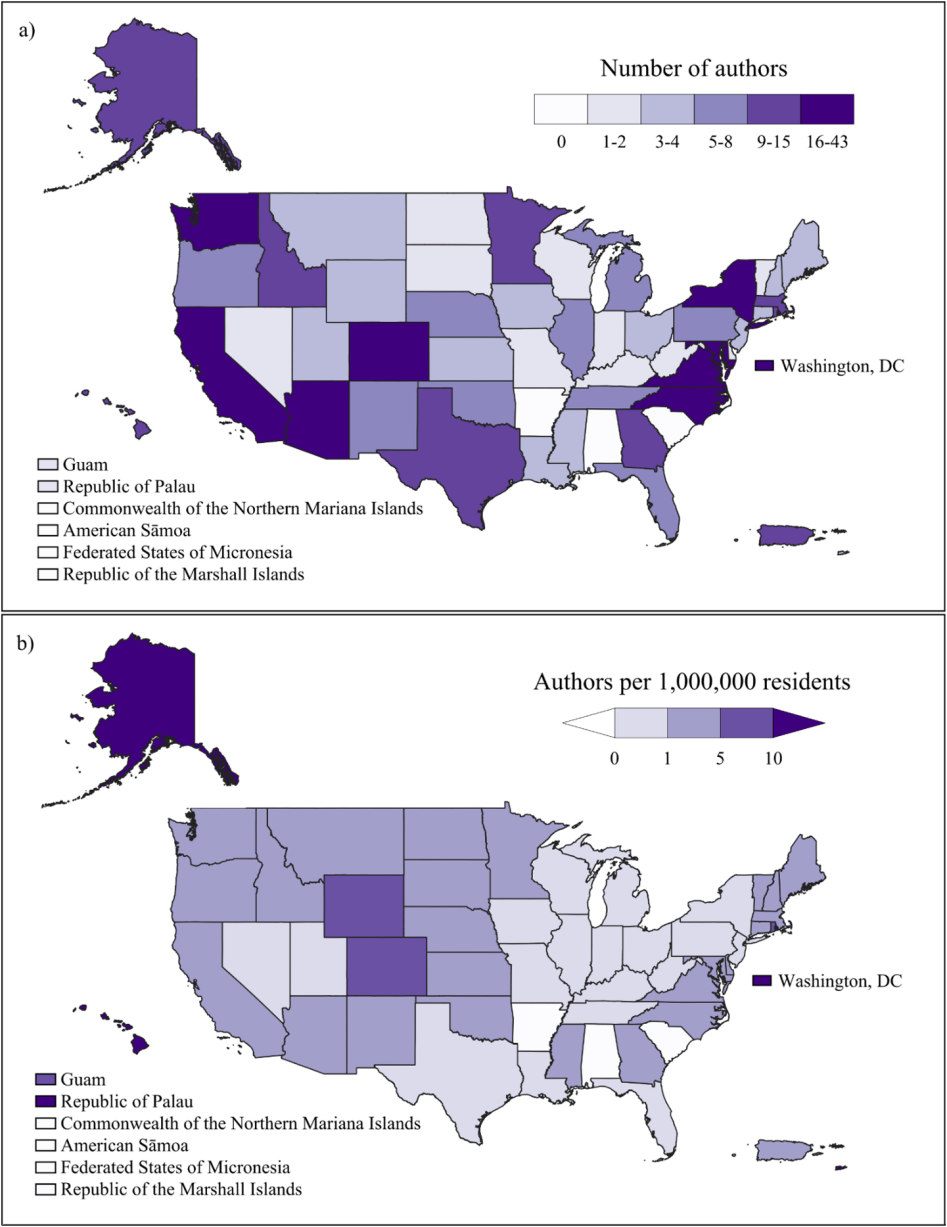
**a** Number of NCA5 authors per state, territory, and US-Affiliated Pacific Island. **b** Number of NCA5 authors per 1,000,000 residents by state, territory, and US-Affiliated Pacific Island. Note: Alaska, Hawai’i, and the U.S. Caribbean islands are not shown to scale. The US-Affiliated Pacific Islands are not shown on the map but are listed. Boundaries: US Census Resident Population: States, Puerto Rico, Guam, and United States Virgin Islands (US Census Bureau 2020) and Republic of Palau Office of Planning and Statistics (2022)

Compared to earlier NCA efforts, NCA5 increased the diversity of its pool of contributors, which included more than 500 authors, more than 200 technical contributors, and 41 review editors. Figure 1 shows the geographic distribution of author affiliations. The largest number of authors was based in California (43), followed by Colorado (30), Washington, DC (29), Maryland (26), and North Carolina (26). North Dakota, West Virginia, Kentucky, Guam, and Palau had the lowest number of authors, with one author each (Fig. 1a). Palau, US Virgin Islands, Washington, DC, Alaska, and Hawai’i had the highest number of authors relative to their populations (Fig. 1b). There were no authors from the states of Alabama, Arkansas, South Carolina, although technical contributors were invited from all US states and territories.

In terms of geographic representation, as defined by the NCA5 regions, the greatest concentration of authors was from the Northeast (120 authors), followed by the Southwest (102 authors) and Southeast (78 authors). The lowest concentrations were from Hawai’i and US-Affiliated Pacific Islands (17 authors), Northern Great Plains (17 authors), and Alaska (12 authors). The number of authors from outside of the contiguous US (OCONUS) dropped off significantly outside of their respective regional chapters.

**Fig. 2:**
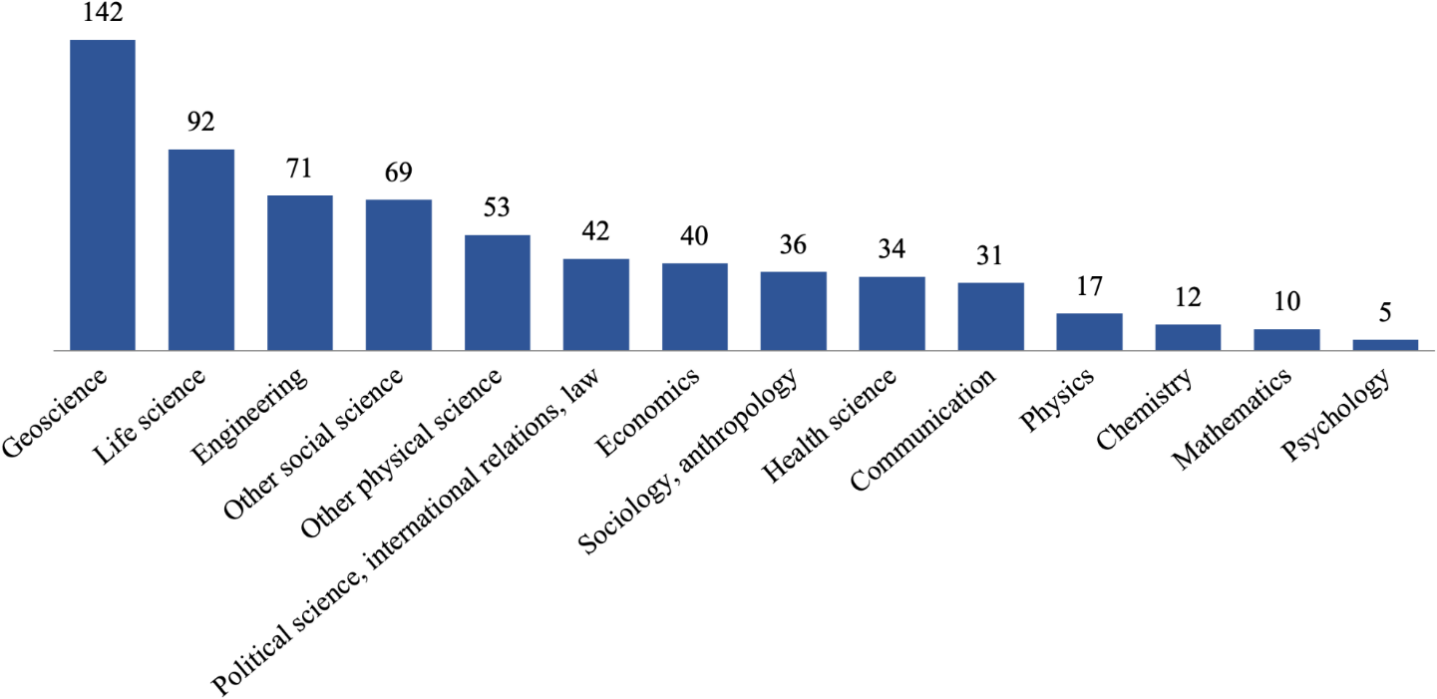
NCA5 author composition by discipline. Note that authors were allowed to select multiple areas of expertise

On the whole, NCA5 tried to include scholars who were leaders in the geophysical and social aspects of climate science. Some chapters — such as the Hawai’i and U.S.-Affiliated Pacific Islands chapter — involved cultural practitioners. USGCRP implemented a survey to catalogue the author team composition in late 2021, resulting in 471 responses. The results show that authors self-identified with a range of disciplines (Fig. 2). While there has been progress in increasing social science representation since NCA4 (Maxwell et al. 2022), the majority of authors still come from geophysical science disciplines. Despite this, some chapters — such as the Ecosystems, Ecosystem Services, and Biodiversity chapter — were able to increase representation of social sciences by drawing on a chapter lead with expertise in governance, sociology, and economics.

**Fig. 3:**
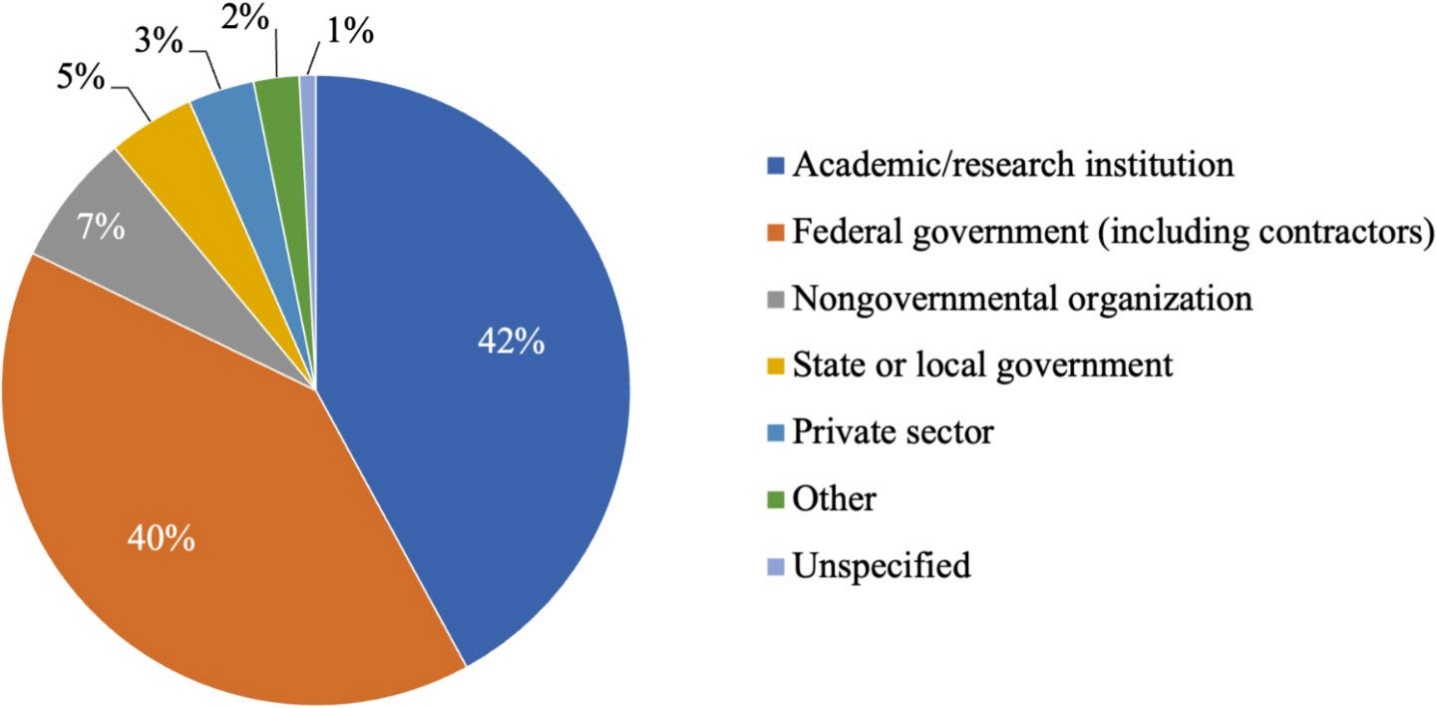
NCA5 author composition by affiliation

Among survey respondents, there were roughly equal numbers of authors from academic institutions and the federal government (including contractors), followed by much smaller proportions from NGOs, state and local governments, and the private sector (Fig. 3).

Many NCA5 chapters relied on technical contributors to broaden representation, source materials, and assessment methods. For example, the Hawai’i and US-Affiliated Pacific Islands chapter included 41 technical contributors who participated in brainstorming sessions that collected written chapter content and helped shape the chapter outline. Including a breadth of technical contributors allowed the chapter to gain important insights from experts who were unable to dedicate the time needed to participate as authors. Contributors included not only research scientists, but also county- and state-level staff, NGOs, and Native Hawaiian- and Indigenous-serving institutions. Several chapters also included students as contributors. For example, the Ecosystems chapter had 12 technical contributors, half of whom were undergraduate and graduate students who were participating in climate assessment for the first time.

Although NCA5 made advances in diversifying authors and contributors, participants were still overwhelmingly from academia, with 42% of authors reporting to be affiliated with a higher education institution and 40% with the federal government. While the reasons likely vary among chapters, the combination of credential gatekeeping, gaps in professional networks, and an expectation that time is volunteered likely prevented participation by practitioners. Despite these barriers, the Alaska chapter, for example, included a commercial fisherman and a community organizer as authors, and the chapter lead worked flexibly with their schedules to allow for meaningful contributions.

There were also difficulties in expanding geographic representation of authors and content. Regional chapters covered vast and diverse areas, so not all regions were included equitably in NCA5. Furthermore, authors representing the chapters on Hawai’i and US-Affiliated Pacific Islands and the US Caribbean noted that they felt excluded from national-level assessment efforts. Many of these inequalities can be traced to regional biases in the scientific literature at large. In the end, NCA5 had a number of maps and figures that did not include OCONUS areas, although each of these were accompanied by a sentence in the caption that highlighted data unavailability (Basile et al. 2024). To bring additional emphasis to this issue, both chapters included a text box highlighting how historical under-resourcing and structures rooted in colonialism have contributed to missing data.

### Diversifying knowledge systems and centering justice

NCA5 provided authors with guidance on writing about people or groups of people from the outset. Authors were encouraged, through trainings and webinars, to consider the justice implications of climate impacts. USGCRP also provided guidance on the use of Indigenous Knowledge. Such efforts drew upon multiple formal Tribal consultations hosted by the White House Council on Environmental Quality and the Office of Science and Technology Policy in support of their *Federal Guidance on the Use of Indigenous Knowledge*. This document called for the recognition of oral and written knowledge, innovations, practices, and beliefs developed by Tribes and Indigenous Peoples in federal research, policy, and decision-making. In response, the NCA5 team revised its Information Quality Guidance, which informed the source material that was used in the assessment. NCA5 authors also expanded assessment-specific guidance on *Writing About Indigenous Peoples*, originally drafted for NCA4.

NCA5 authors made use of intensive deliberations on inclusive language. A memorandum developed by USGCRP’s Social Sciences Coordinating Committee, in collaboration with environmental justice experts on NCA5 author teams, provided informal guidance to authors. The memorandum encouraged authors to avoid using deficit framing when referring to groups of people and provided suggestions for emphasizing the societal underpinnings of present inequalities. An example includes utilizing terms like “frontline” or “overburdened” communities, which highlights socially and historically produced patterns of inequality, rather than relying on language that attributes inequalities to deficiencies within communities. Additional guidance was provided by the NCA5 editorial team on preferred language and on writing about groups of people and equity topics. This helped to broaden topical and disciplinary inclusion of diverse knowledge and experiences.

Despite intentional efforts to center justice issues in NCA5, many NCA5 authors found continued use of essentializing language to assess experiences and histories of diverse groups. In particular, the literature on environmental justice and the literature on Indigenous Peoples are not always the same and can vary depending on context. Grouping people together can result in the erasure of their specific histories and present-day realities. Distinguishing between Indigenous and local knowledge allows for recognizing the histories and knowledges of individual groups. For example, African Americans have had a unique historical experience of slavery, racial segregation, persistent discrimination, and land tenure issues. Meanwhile, Indigenous Peoples have a related but separate history of colonialism, forced displacement, and persecution. These histories contribute to different values, understandings, and insights on climate change that risk oversimplification if not represented carefully and with intention.

### Broadening public engagement

The NCA5 process involved several rounds of public review: two rounds of public comment, one intensive period of public engagement workshops, and an extensive series of post-publication webinars, workshops, and conference events (Lustig et al. n.d.).

At the outset of the assessment process, NCA5 leadership focused on broad outreach and engagement to help authors understand topics important to the public and inform the direction of chapter development. During early 2022, author teams hosted 34 public engagement workshops on the intended approach, objectives, and potential content of each chapter. Workshops were held online and each had a combination of plenary and small group discussions. Over 4,000 individuals from all 50 states, Puerto Rico, U.S. Virgin Islands, Guam, and Micronesia registered, with most representing local, state, federal, or Tribal governments (41%), followed by academic institutions (17%) and NGOs (14%). Three additional author-organized workshops were held in early 2022 and 2023 to expand outreach to targeted groups — two focused on youth participation, co-sponsored by the Youth Environmental Alliance in Higher Education network (represented by Colorado State University, Boston University, and Michigan Tech), Northeastern University, and Rutgers University, which reached more than 200 high school and college students, while another focused on environmental justice and was led by the Rising Voices Center for Indigenous and Earth Sciences. Across all of these events, NCA5 authors interacted directly with participants to answer questions about draft content and gaps in focus areas, and raise the public profile of NCA. Most workshop attendees indicated that it was their first time participating in an NCA event, demonstrating the success of these initiatives in reaching new audiences.

In addition to public engagement workshops, USGCRP also released the draft assessment for written public comment several times over the course of the assessment process. NCA5 received approximately 8,000 comments from both the public and by federal agencies– approximately 900 public comments on the annotated outline, 2,700 public comments on the third order draft, and 4,400 agency comments over the various iterations of the document’s development.

Additional inclusion efforts included simultaneous Spanish translation for the US Caribbean workshop, using closed captioning and other accessible features on the online platform, encouraging diverse ways to participate in the workshop (such as chat, survey, and other interactive functions), and organizing multiple workshops during local times and in the evenings. All NCA5 chapters were freely published online with different digital accessibility features including informational handouts and use of alternative text. For the first time, each chapter was also translated into Spanish to increase public accessibility.

One unique outreach effort of the NCA was an open call for art called Art x Climate (Lustig et al. n.d.). USGCRP received over 800 submissions from across the US from youth, professional artists, and hobbyists. The expressions of climate in art ranged in mediums and geographic representations, and included themes of biodiversity loss, environmental injustices, and cultural identity.

## Conclusion

Explicit efforts to enable DEAI can help broaden participation, diversify knowledge, and involve and honor the experiences and knowledge of frontline communities. NCA5 worked to incorporate DEAI in its goals, selection of assessment leadership, training, and engagement processes. Although progress is ongoing, these actions have nevertheless helped the scientific literature included in NCA5 to be more inclusive and comprehensive. Based on experiences from NCA5, we identify the following as important actions that future efforts — including the forthcoming NCA6 — can take to further DEAI goals.

First, future efforts can build on the clear guidelines articulated in NCA5. NCA5 leadership and authors included informal DEAI champions, who advocated for balanced and inclusive criteria for recruiting contributors and seeking representation from different disciplinary, demographic, and geographic communities. For instance, more recent NCA efforts have achieved equal representation of female- and male-identifying authors, although data on LGBTQIA + authors remains scarce. Data on racial and cultural composition among the author teams was also not collected systematically. While students were included as technical contributors, there was no systemic approach to channel the youth voice to chapter authors. Still, DEAI champions helped to mobilize resources to support public outreach efforts. Strong champions also served as mediators in cases where there were disagreements about DEAI. In future assessments, DEAI champions — potentially formalized through a steering committee — could facilitate review processes that focus on DEAI content, support learning between authors, and share knowledge between authors and the public.

Second, future efforts can provide additional spaces where participants not only synthesize science but dialogue and learn from each other. The science on how we conceptualize vulnerability, structural marginalization, racism, and other drivers of inequality is constantly evolving and requires reflection, especially given the role of assessments in informing policymakers on issues critical to human health and wellbeing. In NCA5, webinars, author-led trainings, townhalls at scientific professional societies, public input, and the in-person author meeting provided opportunities for cross-chapter caucuses. These discussions helped to reduce the use of essentializing language and elevate DEAI as a cross-cutting issue to be broached at the outset rather than as an afterthought.

Third, future efforts can creatively use and involve diverse forms of expertise. Given that academic and government experts reflect existing disparities in representation and that not everyone can commit to extended participation, especially for youth and leaders from under-resourced communities, involving people as technical contributors can help supply more forms of knowledge that are excluded from peer-reviewed literature. A commitment to improving representation of authors from or based in rural areas, areas lacking accessible digital services, small and medium cities, OCONUS areas, and frontline communities is also essential (Basile et al. 2024). Achieving representative assessments necessitates long-term funding and resources at the national level to improve data availability from rural, Tribal, and island regions.

Finally, future efforts can help democratize access to scientific knowledge and improve assessments of knowledge relevant to frontline communities by focusing on local and community-based knowledge. Citizen science, for example, can both help to better represent communities and provide crucial knowledge to fill gaps in under-resourced areas. Researchers can work directly with local leaders and organizers to provide examples, figures, quotations, and art to make assessment insights more robust. This may also mean letting community leaders direct the work: conducting and consulting on the research being done to build trust; guiding analysis through nuanced understandings of community, culture, history, and context; creating best practices to inform how other researchers can work in those communities; and pursuing deep engagement to allow communities to see how their knowledge is used and to co-create climate solutions. In all cases, public engagement efforts should be mindful not to overburden the public with requests, and should be strategically planned to minimize assessment fatigue.

Moving forward, concrete actions to further embed DEAI in climate assessment development processes can focus on creating cross-disciplinary linkages; improving geographic representation, including more diverse sources of science; and elevating the experiences of frontline communities. Actions to formalize DEAI through guidance in author recruitment, the peer review process, public engagement, language translation, or task groups to improve author representation can also be promising first steps. The experiences of the NCA demonstrate that a conscientious effort can effectively mainstream DEAI into the science assessment process. While we have specified these suggestions in the context of the American process, similar processes of diversifying participants, knowledge, and research are likely relevant to other countries. These efforts can help guide how to better conduct climate assessments in more inclusive, just, comprehensive ways.

## Data Availability

All data and materials for the Fifth National Climate Assessment is publicly available via https://nca2023.globalchange.gov.
